# Supplementary material for: Causal Relationship between Aging and Anorexia Nervosa: A White-Matter-Microstructure-Mediated Mendelian Randomization Analysis
Source: Biomedicines. 2024 Aug 16;12(8):1874. doi: 10.3390/biomedicines12081874 (PMC11351342; doi:10.3390/biomedicines12081874)
Supplement: Supplementary file 1 [file biomedicines-12-01874-s001.zip › Supplementary Figure/Figure S4/Singe SNP plot of MR analysis for TL to ubm-b-1790.pdf]

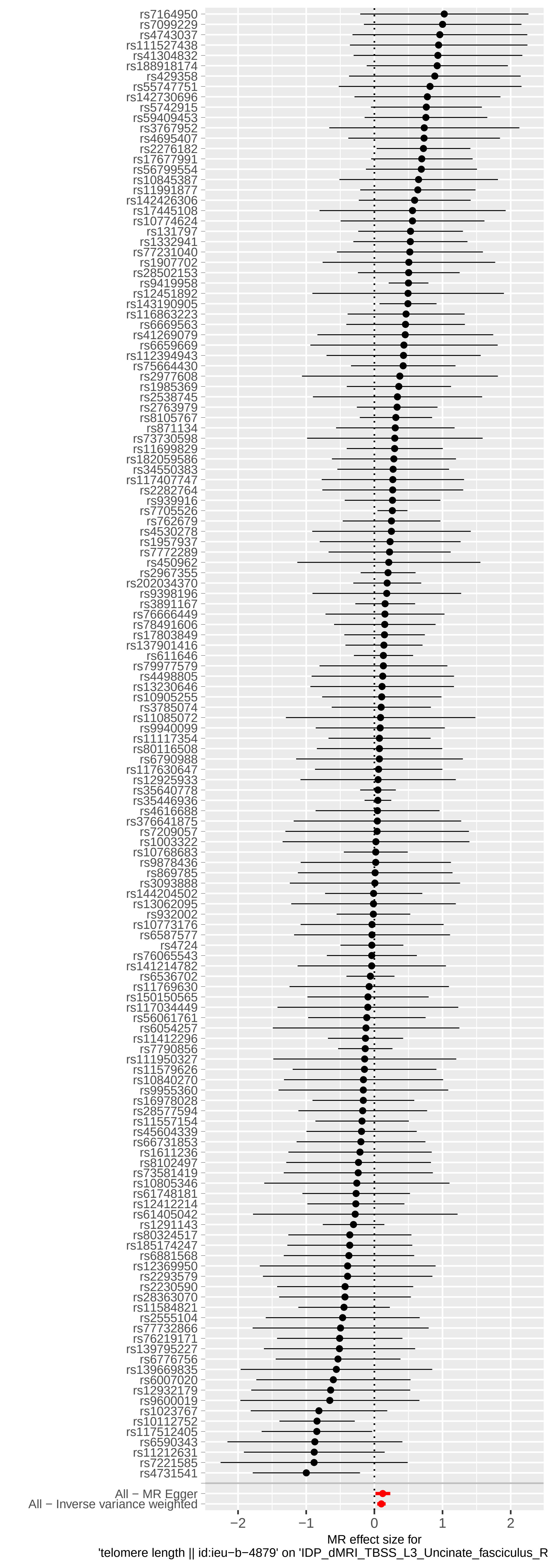
rs7164950
rs7099229
rs4743037
rs111527438
rs41304832
rs188918174
rs429358
rs55747751
rs142730696
rs5742915
rs59409453
rs3767952
rs4695407
rs2276182
rs17677991
rs56799554
rs10845387
rs11991877
rs142426306
rs17445108
rs10774624
rs131797
rs1332941
rs77231040
rs1907702
rs28502153
rs9419958
rs12451892
rs143190905
rs116863223
rs6669563
rs41269079
rs6659669
rs112394943
rs75664430
rs2977608
rs1985369
rs2538745
rs2763979
rs8105767
rs871134
rs73730598
rs11699829
rs182059586
rs34550383
rs117407747
rs2282764
rs939916
rs7705526
rs762679
rs4530278
rs1957937
rs7772289
rs450962
rs2967355
rs202034370
rs9398196
rs3891167
rs76666449
rs78491606
rs17803849
rs137901416
rs611646
rs79977579
rs4498805
rs13230646
rs10905255
rs3785074
rs11085072
rs9940099
rs11117354
rs80116508
rs6790988
rs117630647
rs12925933
rs35640778
rs35446936
rs4616688
rs376641875
rs7209057
rs1003322
rs10768683
rs9878436
rs869785
rs3093888
rs144204502
rs13062095
rs932002
rs10773176
rs6587577
rs4724
rs76065543
rs141214782
rs6536702
rs11769630
rs150150565
rs117034449
rs56061761
rs6054257
rs11412296
rs7790856
rs111950327
rs11579626
rs10840270
rs9955360
rs16978028
rs28577594
rs11557154
rs45604339
rs66731853
rs1611236
rs8102497
rs73581419
rs10805346
rs61748181
rs12412214
rs61405042
rs1291143
rs80324517
rs185174247
rs6881568
rs12369950
rs2293579
rs2230590
rs28363070
rs11584821
rs2555104
rs77732866
rs76219171
rs139795227
rs6776756
rs139669835
rs6007020
rs12932179
rs9600019
rs1023767
rs10112752
rs117512405
rs6590343
rs11212631
rs7221585
rs4731541
All − MR Egger
All − Inverse variance weighted
−2
−1
0
1
2
MR effect size for
'telomere length || id:ieu−b−4879' on 'IDP_dMRI_TBSS_L3_Uncinate_fasciculus_R
